# Antibody Responses are Sustained 2 Years Post-Mpox Infection but not Following Modified Vaccinia Ankara–Bavarian Nordic Vaccination

**DOI:** 10.1093/ofid/ofaf536

**Published:** 2025-08-30

**Authors:** Joanne Byrne, Alejandro Garcia-Leon, Aisling Murphy, Gurvin Saini, Ishan Banik, Alan Landay, Liem Binh Luong Nguyen, Stefano Savinelli, Cathal O’Broin, Mary Horgan, Christine Kelly, Carlos Mejia-Chew, Corinna Sadlier, Eoghan de Barra, Jane A O’Halloran, Virginie Gautier, Patrick W G Mallon, Eoin R Feeney

**Affiliations:** Centre for Experimental Pathogen Host Research (CEPHR), University College Dublin, Dublin 4, Ireland; Department of Infectious Diseases, St Vincent's University Hospital, Dublin 4, Ireland; Centre for Experimental Pathogen Host Research (CEPHR), University College Dublin, Dublin 4, Ireland; Department of Infectious Diseases, St Vincent's University Hospital, Dublin 4, Ireland; Centre for Experimental Pathogen Host Research (CEPHR), University College Dublin, Dublin 4, Ireland; School of Medicine, University College Dublin, Dublin 4, Ireland; Department of Medicine, University of Texas Medical Branch, Galveston, Texas, USA; Institut Pasteur, Université Paris Cité, Paris, France; CIC Cochin Pasteur, Assistance Publique–Hôpitaux de Paris, Paris, France; Centre for Experimental Pathogen Host Research (CEPHR), University College Dublin, Dublin 4, Ireland; Department of Infectious Diseases, St Vincent's University Hospital, Dublin 4, Ireland; Centre for Experimental Pathogen Host Research (CEPHR), University College Dublin, Dublin 4, Ireland; Department of Infectious Diseases, St Vincent's University Hospital, Dublin 4, Ireland; School of Medicine, University College Dublin, Dublin 4, Ireland; Department of Infectious Diseases, Mater Misericordiae University Hospital, Dublin 7, Ireland; Centre for Experimental Pathogen Host Research (CEPHR), University College Dublin, Dublin 4, Ireland; Department of Infectious Diseases, Mater Misericordiae University Hospital, Dublin 7, Ireland; Centre for Experimental Pathogen Host Research (CEPHR), University College Dublin, Dublin 4, Ireland; Department of Infectious Diseases, Mater Misericordiae University Hospital, Dublin 7, Ireland; Department of Infectious Diseases, Cork University Hospital, Co Cork, Ireland; Department of Infectious Diseases, Beaumont Hospital, Dublin 9, Ireland; Department of International Health and Tropical Medicine, Royal College of Surgeons in Ireland, Dublin, Ireland; Centre for Experimental Pathogen Host Research (CEPHR), University College Dublin, Dublin 4, Ireland; Department of Infectious Diseases, St Vincent's University Hospital, Dublin 4, Ireland; Centre for Experimental Pathogen Host Research (CEPHR), University College Dublin, Dublin 4, Ireland; Centre for Experimental Pathogen Host Research (CEPHR), University College Dublin, Dublin 4, Ireland; Department of Infectious Diseases, St Vincent's University Hospital, Dublin 4, Ireland; Centre for Experimental Pathogen Host Research (CEPHR), University College Dublin, Dublin 4, Ireland; Department of Infectious Diseases, St Vincent's University Hospital, Dublin 4, Ireland

**Keywords:** antibodies, immune responses, monkeypox virus, Mpox, MVA–BN vaccine

## Abstract

**Background:**

Clade IIb mpox cases have declined globally, likely due to behavioral changes alongside vaccine- and infection-induced immunity. However, infections in vaccinated individuals raise concerns about immunity durability. We compared the longevity of antibody responses following mpox infection and modified vaccinia Ankara–Bavarian Nordic (MVA–BN) vaccination.

**Methods:**

In a multicenter, prospective cohort, we measured plasma IgG titers to vaccinia virus (VACV) B5 antigen in adults with prior mpox, MVA–BN vaccination, and historical controls, sampled up to 2 years postexposure. Receiver operating characteristic analysis determined the seropositivity threshold. Generalized additive mixed models compared antibody kinetics, and logistic regression identified factors associated with seropositivity. The results are median (interquartile range) unless specified.

**Results:**

A total of 122 vaccinated participants (100% male, aged 36 [32.5–43.5], 25% people with HIV [PWH]) were sampled at 22.0 (20.0–23.5) months post-MVA–BN vaccination, 72 of whom had a paired sample 12.5 (8.0–15.5) months prior, alongside 13 participants post-mpox (100% male, aged 32.5 [30.5–40], 23% PWH) sampled 25.0 (22.5–29.0) months postinfection, 12 with a paired sample 12.5 (8.5–15.5) months prior. At follow-up, 85% (11/13) of the post-mpox group remained seropositive, versus 32% (39/122) of the vaccinated group. Predicted geometric-mean anti-VACV-B5 titers fell below the seropositivity threshold at 15.5 (95% confidence interval [CI]: 13.0–19.5) months postvaccine. PWH had significantly lower odds of retaining seropositivity (odds ratio: 0.18; 95% CI: .04–.60; *P* = .01).

**Conclusions:**

Antibody titers declined more rapidly postvaccination than post-mpox, with most vaccinated recipients, particularly PWH, losing seropositivity at 2 years. How these data relate to reinfection risk or the need for boosters remains to be determined.

## Teaser

In a multicenter prospective cohort study with 2 years follow-up, anti-VACV-B5 IgG remained detectable in 85% at 2 years after mpox infection compared with 32% after modified vaccinia Ankara–Bavarian Nordic vaccination. Antibody titers declined significantly postvaccination, with modeling predicting loss of seropositivity by 15.5 months (95% confidence interval: 13.0–19.5). By 2 years, most vaccinated participants, particularly people with HIV, no longer demonstrated detectable responses, highlighting waning humoral immunity and the need to evaluate correlates of protection and potential booster strategies.

Mpox infections, caused by the monkeypox virus (MPXV), remain a pressing global public health concern. In May 2022, an unprecedented outbreak emerged characterized by sustained autochthonous transmission of clade IIb MPXV in nonendemic regions. This prompted the World Health Organization (WHO) to declare mpox a Public Health Emergency of International Concern (PHEIC) in July 2022 [1]. To date, over 100 000 cases of clade IIb mpox have been reported, with the majority associated with sexual transmission amongst gay and bisexual men who have sex with men (gbMSM) [2]. While the clade IIb outbreak has declined, secondary to behavioral modification and vaccination, a more recent outbreak of clade Ib MPXV in Central Africa continues, which led the WHO to declare a second PHEIC in August 2024 [3].

MPXV belongs to the *Orthopoxvirus* (OPXV) genus, which also encompasses variola virus, the causative agent of smallpox, and vaccinia virus (VACV), the virus used in smallpox vaccination. In the context of the 2022 PHEIC a third-generation smallpox vaccine, modified vaccinia Ankara–Bavarian Nordic (MVA–BN), received emergency use authorization for mpox preexposure and postexposure prophylaxis in Europe. This decision was based on successful protection against infectious challenge in non-human primates [4] and immunogenicity data from phase I–III clinical trials [5–7], rather than real-world efficacy data. Unlike first- and second-generation smallpox vaccines, MVA–BN is nonreplicating and can be safely administered to immunocompromised individuals. Randomized controlled trials (RCTs) have shown that people with HIV (PWH) elicit a humoral immune response to MVA–BN comparable with that observed in people without HIV (PWoH) [8, 9].

A meta-analysis has estimated that 2 doses of MVA–BN provide 81.8% efficacy (95% confidence interval [CI]: 65.0%–89.1%) in preventing mpox infection [10]. Previous studies have also shown that IgG titers against VACV correlate with VACV neutralization in vitro [9, 11] and reported a positive correlation between vaccine effectiveness and VACV-B5-binding antibody titers [10]. While immunological memory to second-generation live smallpox vaccines persists for decades [11], the durability of antibody responses to MPXV following MVA–BN vaccination remains uncertain. Emerging evidence indicates that circulating and neutralizing antibodies induced by MVA–BN vaccination may wane significantly within a year postvaccination, raising concerns about the long-term durability of protection [12–14]. Additionally, increasing reports of breakthrough mpox cases in vaccinated individuals have prompted discussions on the potential need for “booster” doses [15, 16]. However, data on antibody persistence beyond 1-year postvaccination remain limited.

Long-term immunity following mpox infection is similarly poorly understood, with most insights extrapolated from smallpox, which confers lifelong immunity [17]. While lifelong immunity to Mpox postinfection has been presumed, sporadic cases of reinfection have been reported [15]. Observational studies suggest that antibody responses following mpox infection are significantly higher than those induced by vaccination [14, 18, 19], but the durability and trajectory of these responses over time remain unclear.

Complicating the study of mpox humoral immunity is the absence of a standardized approved serological assay. This challenge is amplified by differences between vaccine- and infection-induced antigenic targets, as well as extensive cross-reactivity within the OPXV genus. Through examination of a large number of OPXV antigenic targets, we previously identified IgG targeting the VACV-B5 antigen as the optimal marker for quantifying immunity induced by both vaccination and natural infection [14].

Understanding the durability of immune responses following both vaccination and infection is critical for guiding public health strategies, particularly for vulnerable or immunocompromised subpopulations. This includes optimizing vaccine schedules, determining the need for and timing of booster doses, and identifying populations at greater risk of waning immunity. To address these gaps, we leveraged a validated serological assay to comprehensively assess antibody responses up to 2 years postinfection and vaccination, to map long-term immunogenicity following mpox vaccination and infection, and to identify factors influencing antibody persistence.

## METHODS

### Study Design and Participants

The All Ireland Infectious Diseases (AIID) Cohort Study is a prospective, multicenter, observational study recruiting individuals attending hospital with issues pertaining to infectious diseases (approved by the National Research Ethics Committee in Ireland, reference 20-NREC-COV-056). Adult (≥18 years) participants provide written, informed consent for collection of clinical data and blood samples for biobanking (stored at −80 °C). For this analysis, AIID cohort participants with a history of MVA–BN vaccination or PCR-confirmed MPXV clade IIb disease were included if blood samples were available at least 18 months postcompletion of vaccination or infection. Earlier paired samples, if available, were also included for analysis. Participants included in this analysis were sampled between October 2022 and December 2024. Vaccinated participants received 2 doses of the MVA–BN vaccine (JYNNEOS®), 4 weeks apart, according to national immunization guidelines [20]. Dates of MVA–BN vaccination were collected and verified for all participants using medical records and vaccination certificates. Time since vaccination was calculated in months from the second dose or completion of the vaccine schedule. Data were collected at the time of sample collection on HIV status and CD4^+^ T-cell count. We recorded self-reported participant race and prior smallpox vaccination status using specific cutoffs derived from public health data where participants born before 1972 were presumed to have received childhood smallpox vaccination, and those born after the cessation of smallpox vaccine in 1972 in Ireland or 1989 in the Americas [21, 22] were presumed unvaccinated. Participants born between 1973 and 1989 in the Americas were categorized as having unknown smallpox vaccination status given the regional heterogeneity in vaccination practices during that period [22].

### Electrochemiluminescence Assay

A quantitative electrochemiluminescence (ECL) assay was used to measure antibodies to the VACV-B5 antigen (Sino Biological, Beijing, China), described in detail in the Supplementary methods. A 7-point standard curve was constructed using Non-WHO Reference Material (Working Standard Working reagent for anti-monkeypox antibodies NIBSC code: 22/218). Plates were analyzed using the MESO QuickPlex SQ 120 and MSD Discovery Workbench Software Version 4.0. To determine quantitative antibody concentrations, a conversion equation derived from the linear portion of the standard curve was applied to convert the ng/mL ECL reading into arbitrary units/mL (AU/mL), with results reported in AU/mL.

### Statistical Analysis

Statistical analyses were designed to evaluate both binary seropositivity outcomes and continuous antibody titers in a prospective cohort setting. The seropositivity threshold was determined using receiver operating characteristic (ROC) curves, with the optimal cutoff defined using the Youden Index and area under the curve (AUC), based on true positive (postinfection and postvaccination) and negative control samples from a previous study [17]. We first assessed factors associated with seropositivity at long-term follow-up. Univariate analyses were conducted using the χ^2^ test for categorical variables and the Mann–Whitney U test for continuous variables. Variables with *P* < .10 in univariate testing were included in multivariable logistic regression models. Logistic regression was used to identify independent predictors of seropositivity at follow-up. Sample size calculations were based on previously reported seropositivity rates 1-year postexposure [14], estimating that 120 vaccinated and 12 infected participants would provide >75% power to detect a 38% absolute difference in seropositivity at 2 years (*α* = .05).

To evaluate changes in antibody titers over time, we used generalized additive mixed models, which allow for nonlinear relationships between time since exposure and antibody levels. These models included time as a spline term with a Gaussian distribution, individual participants as random intercepts to account for repeated measures, and an autocorrelation structure to adjust for temporal clustering. To further assess antibody decay, we used linear mixed-effects models (LMMs) with log-transformed IgG titers as the outcome, incorporating HIV status and time since vaccination or infection as fixed effects. Models were adjusted for age and race and included participant ID as a random intercept to account for within-subject correlation. All statistical analyses were performed using R software (version 4.4.1).

## RESULTS

### Study Population

A total of 122 participants were sampled at 22.0 (20.0–23.5) months post-MVA–BN vaccination (72 of whom had a paired sample 12.5 [8.0–15.5] months prior), alongside 13 participants sampled at 25.0 (22.5–29.0) months post-mpox infection (12 with a paired sample 12.5 [8.5–15.5] months prior). All participants with a history of mpox were unvaccinated. Participant clinical characteristics were similar across both groups (Table 1). All participants were assigned male sex at birth, reflecting the population at risk of this outbreak. For the vaccine group, the median (interquartile range [IQR]) age was 36 (33–44) years, 113/122 (93%) were of Caucasian race, and 30/122 (25%) were PWH. In the mpox group, median (IQR) age was 33 (31–40) years, all were of Caucasian race, and 3/13 (23%) were PWH. All PWH in both groups were receiving antiretroviral therapy and had an undetectable viral load (<50 copies/mL). The majority of PWH (27/30 in the vaccine group and 3/3 of the infection group) had a CD4^+^ T-cell count >350 cells/mm^3^ with a median (IQR) CD4^+^ T-cell count at time of assessment of 664 cells/mm^3^ (581–783). The analysis also included 57 previously confirmed positive samples from participants sampled at a median (IQR) of 8 [5–11] months postinfection or vaccination, alongside 63 true negative control samples from participants who were gbMSM without a history of mpox infection or vaccination [14] (Supplementary Table 1).

**Table 1.** Demographics of Study Population

	Vaccination (n = 122)	Infection (n = 13)
**Age, median (IQR)**	36 (32.5–43.5)	32.5 (30.5–40.0)
**Male sex at birth, n (%)**	122 (100%)	13 (100%)
**Race, n (%)**		
Caucasian	113 (93%)	13 (100%)
Asian	9 (7%)	0 (0%)
**HIV, n (%)**		
People with HIV	30 (25%)	3 (23%)
People without HIV	92 (75%)	10 (77%)
**CD4^+^ T-cell count/mm^3^** ^a^, **median (IQR)**	664 (581–783)	713 (603–867)
**Prior smallpox vaccine, n (%)**		
Yes	14 (11%)	0 (0%)
No	96 (79%)	11 (85%)
Unknown	12 (10%)	2 (15%)
**Months from exposure** ^b^ **(visit 2), median (IQR)**	22.0 (20.0–23.5)	25.0 (22.5–29.0)
**Paired samples**	**(n** **=** **72)**	**(n** **=** **12)**
**Months from exposure** ^b^ **(visit 1), median (IQR)**	10.5 (6.0–13.5)	13.5 (11.5–16.0)
**Difference in months between visit 1 and visit 2, median (IQR)**	12.5 (8.0–15.5)	12.5 (8.5–15.5)

Abbreviation: IQR, interquartile range.

^a^CD4^+^ T-cell count/mm^3^ of people with HIV.

^b^Exposure is defined as date of onset of symptoms of mpox infection or dose 2 of MVA–BN vaccine.

### Determination of Seropositivity Threshold

To establish the optimal threshold for distinguishing seropositive samples (either postinfection or postvaccination) from seronegative samples, we constructed an ROC curve based on VACV-B5 IgG titers. This analysis incorporated the 57 true positive samples and 63 true negative controls (Supplementary Figure 1A). The AUC was 0.955 (95% CI: .922–.987). Using the Youden Index to determine the threshold that maximized sensitivity and specificity, an anti-VACV-B5 IgG titer of 3284 AU/mL was identified as the optimal seropositivity threshold, predicting seropositivity with a sensitivity of 98% (95% CI: 90%–100%) and specificity of 84% (95% CI: 78%–95%; Supplementary Figure 1B).

### Antibody Responses 2 Years Post-Mpox Infection and Vaccination

**Figure 1.**
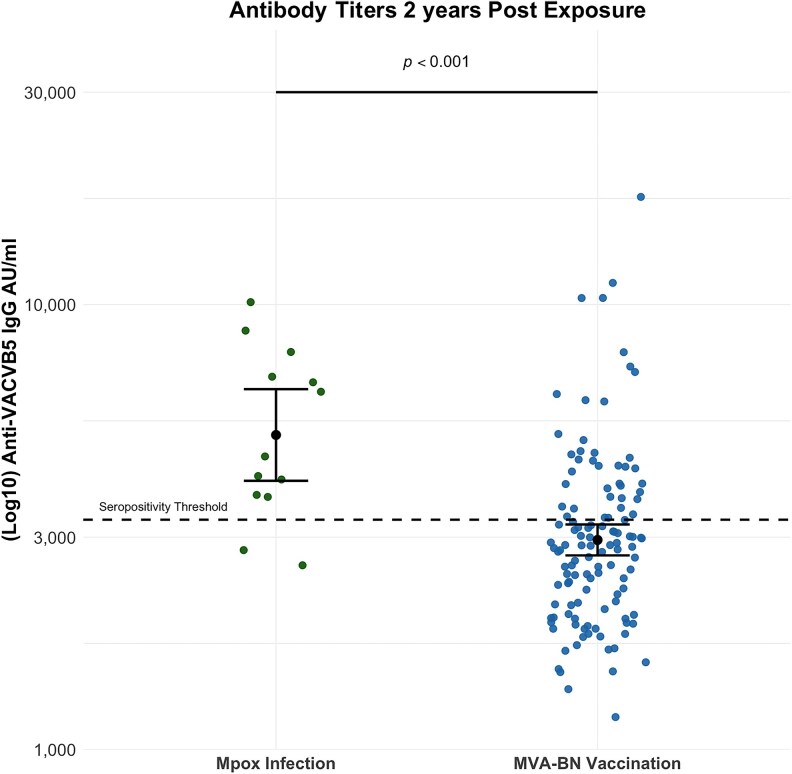
Antibody titers 2 y postexposure. Significantly higher anti-VACV-B5 IgG titers 2 y post-mpox infection (n = 13) and postmodified vaccinia Ankara–Bavarian Nordic vaccination (n = 122). The error bars represent the geometric-mean titer (GMT) and the 95% confidence interval of the GMT for each group. Dashed line represents the seropositivity threshold. *P-*value as per Mann–Whitney U test.

Anti-VACV-B5 IgG titers were significantly higher in participants with a history of mpox infection, sampled at a median of 25.0 (22.5–29.0) months postinfection (geometric-mean titer [GMT]: 5092 AU/mL; 95% CI: 4017–6454), compared with those in the vaccine group, sampled at a median of 22.0 (20.0–23.5) months postvaccination (GMT: 2958 AU/mL; 95% CI: 2731–3205; *P* < .001) (Figure 1). Notably, 85% (11/13) of participants in the mpox group remained seropositive at 2 years compared to only 32% (39/122) of participants in the vaccine group who maintained seropositivity at a similar time point (*P* < .001).

Demographics and clinical characteristics of participants in the vaccine group above and below the seropositivity threshold at 2 years postexposure are summarized in Table 2. Clinical characteristics were similar between groups with the exception of HIV status. The seropositive group had a significantly lower prevalence of PWH (7%, 3/39) compared with the seronegative group (33%, 27/92; *P* = .01). At 2 years, PWH had lower antibody titers (GMT: 2645 AU/mL; 95% CI: 2321–3015) than PWoH (GMT: 3068 AU/mL; 95% CI: 2786–3379), although this difference was not statistically significant (*P* = .08). Furthermore, anti-VACV-B5 titers did not correlate with CD4^+^ T-cell count (rho = 0.15, *P* = .42). However, PWH were 82% less likely to maintain seropositivity postvaccination (odds ratio: 0.18; 95% CI: .04–0.60; *P* = .01).

**Table 2.** Demographics and Clinical Characteristics of Participants Above and Below the Seropositivity Threshold 2 Years Postvaccination

	Above seropositivity threshold (n = 39)	Below seropositivity threshold (n = 83)	*P-*Value
**Age, median (IQR)**	36.0 (32.0–40.0)	36.5 (32.5–44.0)	.21*
**Male sex at birth, n (%)**	39 (100%)	83 (100%)	NA
**Race, n (%)**			
Caucasian	37 (95%)	76 (92%)	.78**
Asian	2 (5%)	7 (8%)	
**HIV, n (%)**			
People with HIV	3 (8%)	27 (33%)	.01**
People without HIV	36 (92%)	56 (67%)	
**CD4^+^ T-cell count/mm^3^** ^a^ **, median (IQR)**	638 (586–710)	666 (582–787)	.63*
**Prior smallpox vaccine, n (%)**			
Yes	2 (5%)	12 (14%)	.12**
No	35 (90%)	61 (73%)	
Unknown	2 (5%)	10 (12%)	
**Months from exposure** ^b^ **(visit 2), median (IQR)**	21.5 (21.0–23.5)	21.5 (20.0–23.0)	.30*

Abbreviations: IQR, interquartile range; NA, not applicable.

^a^CD4^+^ T-cell count/mm^3^ of people with HIV.

^b^Exposure is defined as date of onset of symptoms of mpox infection or dose 2 of MVA–BN vaccine.

^*^ *P-*value derived from Mann–Whitney U test.

^**^ *P-*value derived from χ^2^ test.

### Antibody Kinetics Over Time

**Figure 2.**
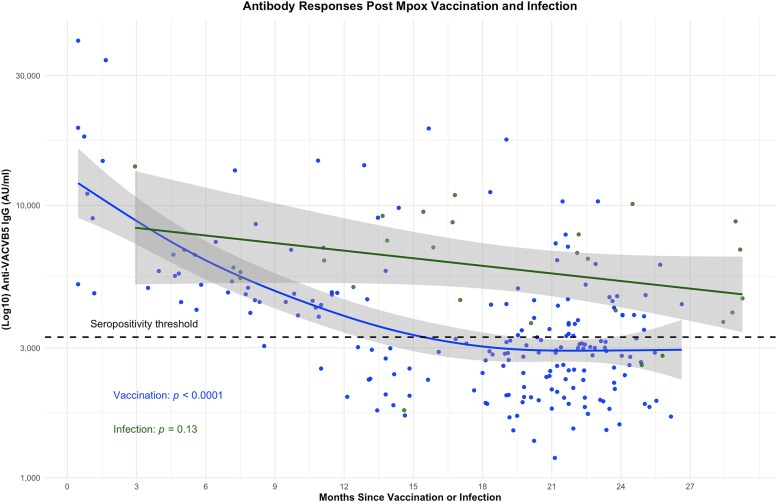
Antibody responses post-mpox vaccination and infection. Change in VACV-B5 IgG titers over 2 y following mpox infection or modified vaccinia Ankara–Bavarian Nordic vaccination. Scatter plots display individual antibody titers, with superimposed generalized additive mixed model (GAMM) curves fitted using a Gaussian link function. Time since symptom onset or vaccination was modeled as a spline. Antibody titers declined significantly postvaccination (*P* < .0001) but remained stable postinfection (*P* = .13). The dashed line represents the seropositivity threshold. The predicted mean anti-VACV-B5 titer falls below this threshold at 15.5 m (95% confidence interval: 13.0–19.5) postvaccination. The *P*-values represent the significance of the relationship between time and antibody titers as estimated by GAMM.

We evaluated the kinetics in antibody responses over time postinfection and vaccination using all available samples: 194 from 122 participants sampled post-mpox vaccination and 25 samples from 13 participants sampled post-mpox infection. Antibody titers declined significantly postvaccination (*P* ≤ .0001), with the mean predicted anti-VACV-B5 titers following MVA–BN vaccination falling below the seropositivity threshold at 15.5 (95% CI: 13.0–19.5) months postvaccination (Figure 2). In contrast, antibody titers did not significantly change over time postinfection (*P* = .15) and the mean predicted anti-VACV-B5 titers, including the 95% CI following mpox infection remained above the seropositivity threshold throughout follow-up (Figure 2). However, the small number of participants in the mpox group may limit the precision of these estimates. Overall, the aggregated predicted mean anti-VACB5 titer in the MVA–BN vaccine group (3587 AU/mL) was significantly lower than the mpox group (5805 AU/mL, *P* < .0001) for the duration of follow-up.

### Differential Antibody Decay Following Vaccination by HIV Status

**Figure 3.**
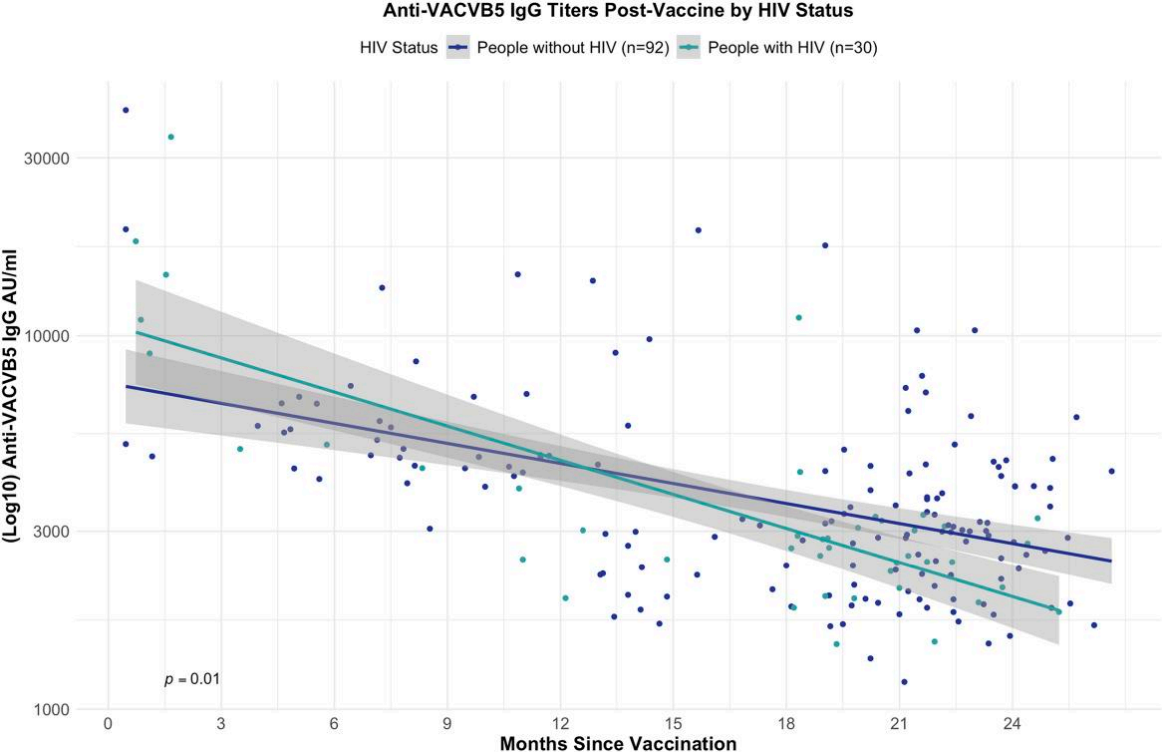
Antibody titers postvaccination by HIV status. Decline in anti-VACV-B5 IgG titers following modified vaccinia Ankara–Bavarian Nordic vaccination stratified by HIV status. Scatter plots display individual antibody titers, with superimposed linear mixed-effects model regression lines and 95% confidence intervals. Titers declined significantly over time in both groups, with a steeper decline observed in people with HIV. The *P*-value (*P* = .01) represents the statistical significance of the association between time since vaccination and antibody titers, adjusted for repeated measures within individuals.

We evaluated the decay of anti-VACV-B5 IgG titers following vaccination over time in participants with and without HIV. In both groups, anti-VACV-B5 IgG titers decreased significantly with time since vaccination (both *P* ≤ .0001), consistent with waning of antibody responses over time postvaccination. An LMM, adjusted for age and race, identified a significant interaction between HIV status and time since vaccination (*P* = .01), indicating a significantly steeper rate of decline in antibody titers over time among PWH compared with PWoH (Figure 3).

## DISCUSSION

This study demonstrated a significant decline in anti-VACV-B5 IgG antibody titers over time following mpox vaccination, while titers remained relatively stable in participants with prior mpox infection. We estimated that the predicted mean antibody titer in vaccinated participants falls below the seropositivity threshold at 15.5 (95% CI: 13.0–19.5) months postvaccination, whereas titers in the mpox-infected group remained above this threshold throughout the 2-year follow-up period. Furthermore, rates of decline in postvaccination antibody titers were more rapid, and seropositivity rates at 2 years significantly lower in PWH at 2 years.

To our knowledge, this is the first study to evaluate circulating antibody responses up to 2 years post-mpox infection and vaccination for mpox prevention. We observed a significant sustained decline in circulating antibody titers postvaccination, with only 32% of vaccinated participants maintaining titers above the seropositivity threshold at 2 years. The MVA–BN vaccine was authorized for mpox prevention under emergency use provisions during the PHEIC, and no RCTs have evaluated its long-term immunogenicity or protective efficacy against mpox infection. Observational studies conducted during the 2022 global outbreak provided initial clinical evidence supporting vaccine effectiveness against mpox, supporting the decision to use these vaccines during the outbreaks [10, 23–25]. However, these studies were limited by short follow-up durations, leaving the potential for waning immunity unaddressed. While recent analyses have reported declining antibody titers following vaccination, follow-up has remained largely restricted to the first year postvaccination [13, 14, 19]. While declining titers raise concerns about reduced protection, the clinical significance remains uncertain, particularly in the absence of a defined correlate of protection. Although our study did not assess anamnestic responses, a robust memory response could mitigate the effect of declining circulating antibodies. A clinical trial assessing antibody responses to an MVA–BN booster 2 years postvaccination for smallpox prevention demonstrated a rapid anamnestic response in neutralizing antibodies following boosting, despite the absence of sustained neutralizing antibody titers prebooster, suggesting the potential for durable immunological memory and recall responses [26]. This raises the possibility that individuals with low or waning titers may still retain effective protection upon re-exposure through a rapid recall response. In the context of mpox prevention, our findings underscore concerns around waning humoral immunity, with only a minority of vaccinees maintaining seropositivity at 2 years. However, without data on memory B- or T-cell responses, the clinical implications of these findings remain uncertain. Future studies should incorporate immune recall assessments to better understand the true durability of vaccine-induced protection and inform booster strategies.

A concerning finding in this study is the differential immunogenicity observed in PWH. Compared with PWoH, PWH were 82% less likely to maintain antibody titers above the seropositivity threshold at 2 years postvaccination, coupled with a significantly accelerated rate of antibody decay. These findings align with prior reports indicating that PWH exhibit lower neutralizing antibody titers 6 months postvaccination compared with PWoH [12]. This may be attributed to residual immune activation, premature cellular senescence, and impaired germinal center responses in PWH [27]. Given that PWH (particularly those with advanced HIV) are disproportionately affected by mpox and are at higher risk of severe outcomes [28], our data add to a growing body of work that suggests tailored or antibody-guided booster vaccination strategies may be required to maintain protection against infection in this vulnerable population.

Historically, live, attenuated vaccines have provided the most robust and durable immunity against viral infection. Using a modified nonreplicating vaccine such as the MVA–BN may elicit a less durable response due to its limited antigenic presentation and reduced stimulation of innate immune pathways. The significant waning of vaccine-induced antibody titers over time raises important questions regarding the optimal timing of vaccine schedules and the potential need for booster doses to sustain protective immunity. Notably, emerging vaccine platforms, such as mRNA-based vaccines, have demonstrated durable long-term immunity for SARS-CoV-2 [29] and are being actively investigated for mpox prevention. One study in non-human primates reported that an mRNA-based mpox vaccine-induced enhanced binding and neutralizing antibody responses compared with MVA–BN, suggesting a potential avenue for improving vaccine durability [30].

In marked contrast to postvaccine immune responses, participants with prior mpox infection maintained significantly higher antibody titers at 2 years, with 85% maintaining titers above the seropositivity threshold. These data build on a previous study reporting significantly higher neutralizing antibodies at 6 months post-mpox infection compared with post-MVA–BN vaccination [12]. Furthermore, that no significant decline in antibody titers was observed postinfection suggests more durable immunity, although small sample size restricts definitive conclusions. This may reflect a broader antigenic exposure and enhanced engagement of both the innate and adaptive immune systems. Natural infection elicits a more expansive humoral response than vaccination, as evidenced by a higher frequency of H3L-specific IgG^+^/CD19^+^ B cells in infected individuals than vaccinated individuals [31]. Similar patterns in influenza immunity suggest that infection, rather than vaccination drives sustained antibody production through robust memory B cell and long-lived plasma cell engagement, which are critical for sustained antibody production [32].

This study has several limitations. At first, the demographic composition was limited to adult participants assigned male sex at birth, reflecting the population most affected during the clade IIb mpox outbreak. All participants with mpox had MPXV clade IIb disease; as such, immune responses to clade Ib—currently responsible for PHEIC in Central Africa—remain uncharacterized in this context. The small sample size in the mpox group limits the precision of the modeled estimates of postinfection antibody kinetics and constrains our ability to evaluate the impact of HIV on infection-induced antibody responses. Additionally, the study population was predominantly of Caucasian race, with no representation from individuals of Black African race. This is a key limitation, particularly given the disproportionate burden of mpox in African populations. We also inferred smallpox vaccination status based on birth year and region, which may have introduced misclassification. Although no subanalysis was conducted based on presumed smallpox vaccination status, this assumption may limit the generalizability of our findings. These gaps highlight the need for more research in vaccine effectiveness in geographically and ethnically diverse populations, which is being addressed in studies such as the Mpox-Vax Afrivac trial (EUCT pending).

Another limitation is the absence of an international standard unit for OPXV binding antibody titers. Our analysis used arbitrary units derived from a 7-point standard curve based on non-WHO reference material, limiting cross-study comparability. Establishing an internationally recognized standard for mpox serological assays is essential to facilitate harmonization of results across laboratories. Although a meta-analysis has reported a positive correlation between vaccine effectiveness and VACV-B5-binding antibody titers [10], the extent to which declining titers correspond to increased risk of breakthrough infection remains unclear, and the protective threshold of antibody required for clinical immunity has yet to be defined. Neutralizing antibody responses were not evaluated, limiting our ability to assess functional correlates of protection. The relationship between binding antibody titers and neutralization capacity is essential to establish their clinical relevance as predictors of protective immunity. In addition, T-cell responses, which may play an important role in protection, were not evaluated. Isolated cases of atypical mpox have been reported in individuals with high antibody titers [33], indicating that antibody titers alone may not fully predict protection and that other components of the immune response, such as T-cell or mucosal immunity, may also play a role. Ongoing studies with longitudinal follow-up, B- and T-cell immune profiling, and clinical endpoints will be essential to fully elucidate the durability and breadth of vaccine-induced immunity.

Despite evidence of declining antibody titers, real-world epidemiological data show that mpox case rates have remained relatively low in the global north up to 2 years postvaccination [2]. This suggests a degree of sustained protection, possibly through sustained cellular immunity or rapid recall response. However, it remains difficult to disentangle the potential contribution of behavioral changes within the population at risk, which may also have influenced transmission dynamics. Finally, any discussion of booster strategies must consider the broader global context. While high-income countries may consider additional doses to sustain protection, access to MVA–BN remains limited in many African countries where mpox is endemic and the PHEIC continues. The potential redirection of limited vaccine supplies for preemptive boosting in low-incidence settings therefore warrants careful consideration.

Despite these limitations, this study provides the first comprehensive evaluation of antibody responses up to 2 years post-mpox infection and vaccination. The observed decline in vaccine-induced antibody titers over time, particularly in PWH, has potential implications for long-term protection and vaccine policy. Given the ongoing PHEIC, the waning of vaccine-induced immunity is concerning and underscores the need for continued research into booster strategies, novel vaccine platforms, and tailored approaches to immunization in vulnerable populations.

## Supplementary Material

ofaf536_Supplementary_Data

## Supplementary Data

Supplementary materials are available at *Open Forum Infectious Diseases* online. Consisting of data provided by the authors to benefit the reader, the posted materials are not copyedited and are the sole responsibility of the authors, so questions or comments should be addressed to the corresponding author.
