# Supplementary material for: Antibody Responses are Sustained 2 Years Post-Mpox Infection but not Following Modified Vaccinia Ankara–Bavarian Nordic Vaccination
Source: Open Forum Infect Dis. 2025 Aug 30;12(9):ofaf536. doi: 10.1093/ofid/ofaf536 (PMC12461842; doi:10.1093/ofid/ofaf536)
Supplement: ofaf536_Supplementary_Data [file ofaf536_supplementary_data.zip › mpox_follow_up_supplementary_CID.docx]

**Supplementary Materials: Antibody Responses are Sustained Two Years Post Mpox Infection but not Following MVA-BN Vaccination**

**Methods:**

**Electrochemiluminescence assay**

A quantitative electrochemiluminescence (ECL) assay was used to measure antibodies to the VACVB5 antigen (Sino Biological, Beijing, China), as previously described in detail elsewhere(1). VACVB5 antigen was selected due to its optimum performance for evaluating antibodies both post infection and vaccination. Briefly, reconstituted antigen was coated onto 96-well high-bind plates (MSD, Rockville, MD) at 5 picomoles per well overnight at 4°C, washed with PBS-Tween (Bio Sciences Ltd., Ireland), blocked with 1% blocker A (MSD) for 120 minutes at room temperature, and then plasma diluted 1:500 in MSD D100 was added. A standard curve was constructed using Non-WHO Reference Material (Working Standard Working reagent for anti-monkeypox antibodies NIBSC code: 22/218) with serial 1:2 dilutions in MSD D100 to create a 7-point standard curve ranging from 1:25 – 1:1600, with 25ul of each standard dilution added per well. Each plate included three biological controls; plasma from a Mpox-infected participant, an MVA-BN vaccinated participant, and a negative control. Following incubation, plates were labelled with MSD SULFO-TAG-labelled anti-human IgG secondary antibody, MSD GOLD read buffer A was added, and plates were analysed using the MESO QuickPlex SQ 120 and MSD Discovery Workbench Software Version 4.0. To determine quantitative antibody concentrations, a conversion equation derived from the linear portion of the standard curve was applied to convert the ng/mL ECL reading into arbitrary units/ml (AU/ml), with results reported in AU/mL.

**Supplementary Table 1**

|  | **True positive (n=57)** | **Negative (n=63)** |
| --- | --- | --- |
| **Age (median (IQR))** | 33.5 (30.0 - 40.5) | 33.5 (31.0 - 38.5) |
| **Male sex at birth (n(%))** | 57 (100%) | 63 (100%) |
| **Race (n(%))**  Caucasian  Asian | 52 (91%)  5 (9%) | 56 (89%)  6 (11%) |
| **HIV (n(%))**  People with HIV  People without HIV | 11 (19%)  46 (81%) | 20 (32%)  43 (68%) |
| **Exposure type (n(%))**  Post MVA-BN Vaccination  Post Mpox Infection | 46 (81%)  11 (19%) | NA |
| **Months from exposure^a^ (median (IQR)** | 8 (5-11) | NA |

**Supplementary Figure 1: Determination of Seropositivity Threshold**

**
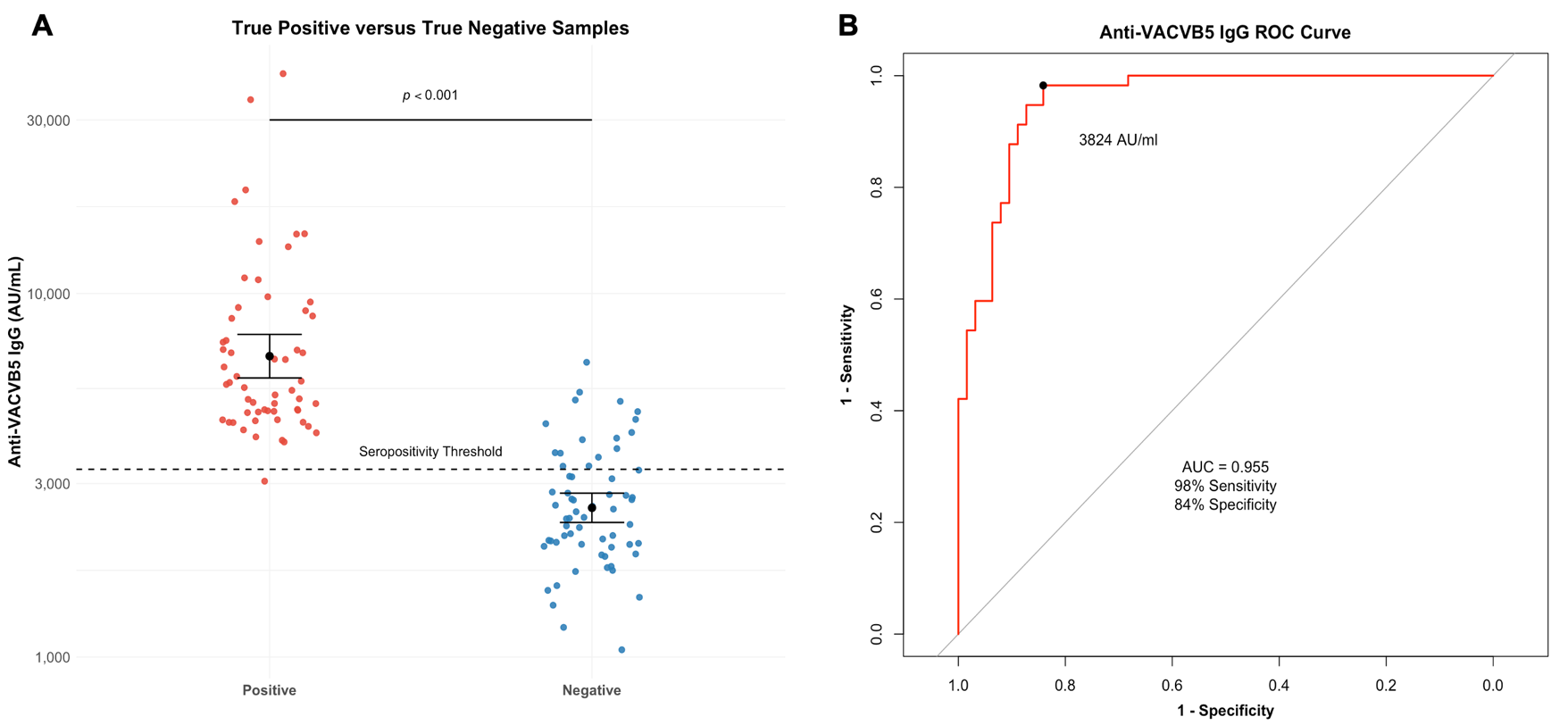
**

A: Anti-VACVB5 IgG titres from true positive samples (n=57) and true negative samples (n=63). The error bars represent the geometric mean titre (GMT) and the 95% confidence interval of the GMT for each group. Dashed line represents the seropositivity threshold. *p* value as per Mann-Whitney U Test.

B: ROC curve for Anti-VACVB5 IgG generated by comparing the true positive samples (n=57) to true negative samples (n=63).

**References**

1. Byrne J, Saini G, Garcia-Leon A, Alalwan D, Doran P, Landay A, et al. Development and validation of a quantitative Orthopoxvirus immunoassay to evaluate and differentiate serological responses to Mpox infection and vaccination. eBioMedicine. 2025;113.
