# Supplementary figures and images for: Antibody Responses are Sustained 2 Years Post-Mpox Infection but not Following Modified Vaccinia Ankara–Bavarian Nordic Vaccination
Source: Open Forum Infect Dis. 2025 Aug 30;12(9):ofaf536. doi: 10.1093/ofid/ofaf536 (PMC12461842; doi:10.1093/ofid/ofaf536)

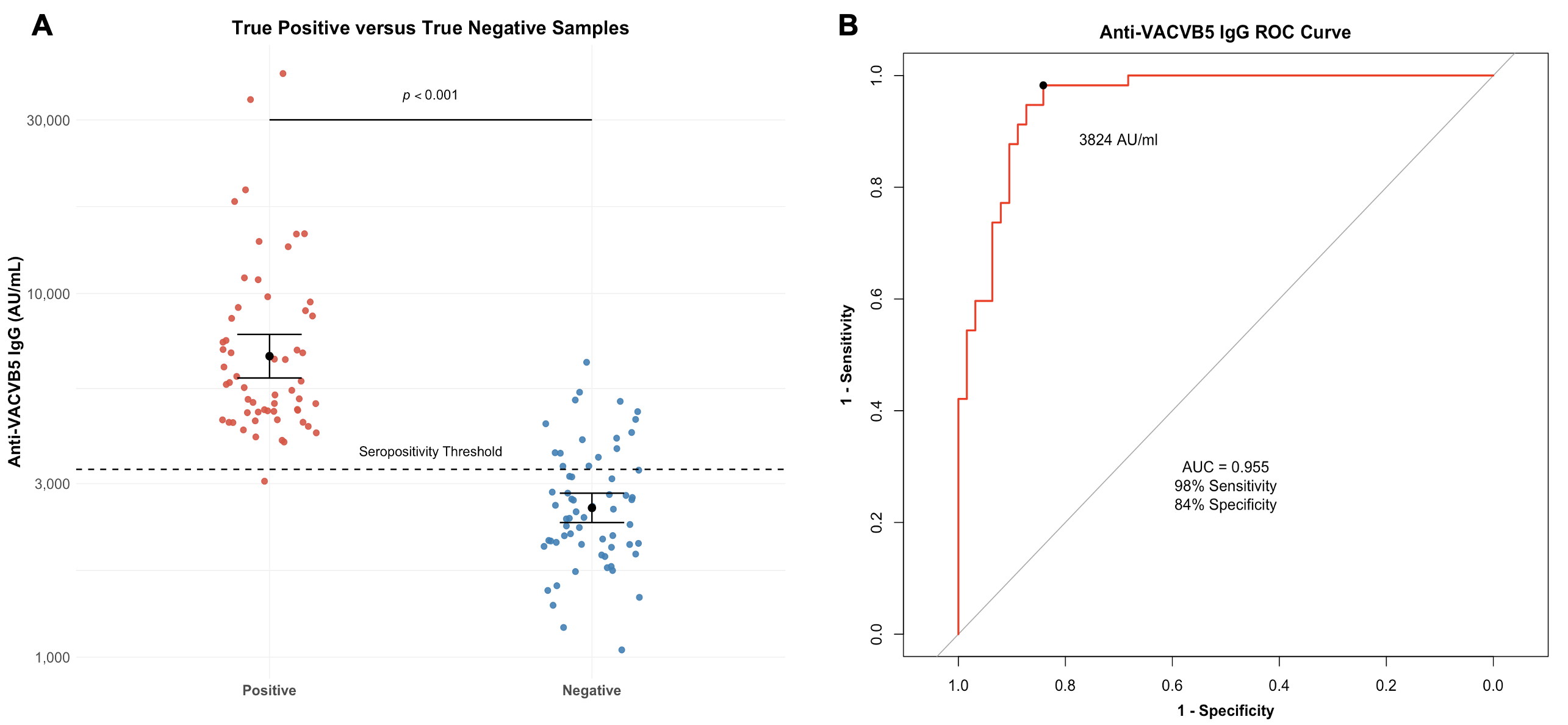

Supplement: ofaf536_Supplementary_Data [file ofaf536_supplementary_data.zip › Supp_Fig_1_300dpi.tiff]
